# Supplementary material for: Are carbon emissions trading and green financial instruments synergistic? -Comprehensive quantitative research based on content analysis
Source: PLoS One. 2024 Mar 7;19(3):e0298601. doi: 10.1371/journal.pone.0298601 (PMC10919601; doi:10.1371/journal.pone.0298601)
Supplement: S1 Data — (ZIP) [file pone.0298601.s001.zip › Data/地级市Z_F工作报告.zip!/╨┬╜«/╬┌┬│─╛╞δ╩╨/└┤╫╘╣1⁄2╓┌║┼í╛┬φ┐╦╩2╛▌═°í┐_2015.docx]

各位代表：

　　现在，我代表市人民政府，向大会报告工作，请予审议，并请各位政协委员、列席代表提出意见。

　　2014年工作回顾

　　过去的一年，是经受严峻考验、克服重重困难、极不平凡的一年。在自治区党委、自治区人民政府和市委的坚强领导下，在市人大、市政协的监督支持下，市人民政府团结带领全市各族干部群众，坚决贯彻落实党的十八届三中、四中全会、第二次中央新疆工作座谈会和自治区党委八届七次、八次全委（扩大）会议以及市委十届七次、十次全委（扩大）会议精神，紧紧围绕社会稳定和长治久安这一总目标和着眼点着力点，坚持稳中求进、改革创新，紧贴民生推进跨越式发展，圆满完成了市十五届人民代表大会第三次会议确定的各项目标任务，为首府社会稳定和长治久安奠定了坚实基础。

　　（一）反恐维稳斗争取得新胜利

　　社会稳控和防范水平全面提升。深入开展严打暴恐专项斗争，及时打掉一批危安暴恐团伙组织，快速果断处置“4·30”、“5·22”严重暴恐案件，有力打击了“三股势力”嚣张气焰。全面强化人员密集场所安全防范等六个方面措施。建立督查整改机制，强化维稳责任落实，及时消除了各种隐患。

　　基层基础进一步巩固。所有社区均达到“十有”条件，工作经费达到25万元以上。完善社区“四化”管理，网巡、步巡、车巡相结合的治安防控体系不断健全。扎实开展“访惠聚”活动，切实做好维护稳定和服务群众工作。完善手机用户的短信发布平台，在最短时间内澄清事实真相，驳斥各类谣言，回应市民关切。各族干部群众主动发声亮剑，在暴恐犯罪面前挺身而出，构建起反恐维稳的铜墙铁壁。

　　“去极端化”工作深入推进。制定4个方面41条“去极端化”措施，坚决铲除宗教极端思想滋生土壤。严厉打击“三非”活动，依法查处一批地下讲经点和非法宣传品。将“制止非法宗教活动”和“抵御宗教极端思想”纳入中小学思想教育内容，在全市教育系统开展“讲法治、促团结、防渗透”集中教育。开展“去极端化”宣讲2300余场次，增强了各族群众明辨是非的能力。

　　民族宗教工作明显加强。深入开展马克思主义“五观”、“四个认同”和“三个离不开”宣传教育。启动各民族相互嵌入式小区示范工程，各族群众交流交往不断加强。举办“社区民族团结讲台”活动2800余场，民族团结先进事迹巡回报告220余场次；面向流动人口开展文艺展演及文化志愿服务300余场，营造了民族团结的浓厚氛围。依法加强宗教事务管理，严格落实宗教场所“三管一负责”和领导干部联系清真寺制度，扎实开展“两模范”、“双五好”创建活动。

　　（二）经济保持持续快速增长

　　预计实现地区生产总值2510亿元，增长10.5%；全社会固定资产投资1526亿元，增长20%；社会消费品零售总额1070亿元，增长10.3%；外贸进出口总额132亿美元（全口径），增长10%；地方财政收入452.96亿元，增长13%，其中公共财政预算收入340.62亿元，增长12.8%；城镇居民人均可支配收入23755元，增长11.5%；农牧民人均纯收入13335元，增长16%。

　　“五大产业基地”规模不断扩大。经济技术开发区（头屯河区）先进制造业基地、高新技术产业开发区（新市区）高新技术产业基地、甘泉堡经济技术开发区战略性新兴产业基地、会展片区现代服务业基地、南山旅游基地建设加快推进，已入驻世界500强企业20家，中国500强企业66家，产业结构得到进一步优化提升。“五大产业基地”经济总量和财政收入占到全市50%左右，为全市全口径财政收入过千亿元作出积极贡献，已成为首府经济跨越式发展的重要支撑。

　　新型工业化加快推进。完成工业增加值780亿元，增长12%。实施重点工业项目30个，其中战略性新兴产业项目占63.3%。新增高新技术企业36家，实现增加值107.62亿元，增长24%。新疆软件园投入使用，65家企业获批入园。广电网络、中科曙光落户云计算产业园。启动智慧城市建设项目25项。新增国家及自治区企业技术中心6家、产学研联合开发创新示范基地7个、市级创新型企业21家、产业技术创新联盟2个。专利申请量同比增长13.64%，发明专利万人拥有量首超全国平均水平。成功申报国家节能减排财政政策综合示范城市。淘汰落后产能155万吨，万元工业增加值能耗同比下降12.11%，提前完成“十二五”污染物总量减排任务。

　　服务业快速发展。实现服务业增加值1525亿元，增长9.3%。国家服务业综合改革、城市共同配送、农产品流通综合试点城市和电子商务示范基地建设扎实推进，完成乌鲁木齐国际纺织品服装商贸中心总体规划。加快推进会展片区金融城、高铁片区中亚国际商贸金融中心核心区等规划建设步伐，乌鲁木齐陆路港、乌拉泊国际物流基地项目建设进展顺利，万达、宝能、绿地、奥特莱斯等城市综合体项目加快建设。组团参加国内外大型展会17个，主办承办2014年丝绸之路国际食品展览交易会暨第四届中国清真美食文化节、文化创意博览会等展会86个。在第四届中国—亚欧博览会上，乌鲁木齐交易团签约总额达1808亿元，较上届增长20.3%。全年接待国内外游客2014.5万人次，增长3.13%。

　　农业农村工作全面推进。农业产业结构进一步优化，蔬菜生产面积达到28.3万亩，新建温室大棚6000亩、标准化养殖小区60个，新增肉羊（牛）生产能力30万只。农村土地规模化流转11.4万亩，新增农民专业合作社429家、农业产业化龙头企业12家。完成16个乡镇、122个村的规划编制任务，2.8万户“两居”工程全部开工。实施农村安全饮水项目26个，解决5.3万农牧民安全饮水问题。新建农村公路86公里，新建农村班线公交化站点62座。水西沟镇和白杨沟村、东风村分别被评为国家级生态乡镇、自治区级生态村。

　　（三）民生改善取得新成效

　　全面完成10个方面100件实事。市财政用于民生领域的支出301.55亿元，占公共财政预算支出的74.5%，增长15.23%。启动中心城区6个大型农贸市场建设，改建28个标准化集贸市场，在取消早夜市区域新建27个社区蔬菜副食品直销点。加大重点行业及重点民生商品的价格监管和专项整治，投放政府储备肉2787吨、蔬菜5万吨，居民基本生活品价格保持稳定，居民消费价格总水平涨幅为3.2%。加快推进“公交都市”建设，BRT7号线建成运营，新增和更新公交车303辆，新增出租车150辆，新开通（优化）公交线路16条，新增公交站点118个，改造规范公交站点1215个，中心城区公交线路实现智能化调度全覆盖。深入治理交通拥堵，对16条路段车辆违停进行专项治理，新增18条便民停车路段、28条单行道，改造提升背街小巷118条。加强物业市场规范管理，制定物业管理条例实施细则和物业专项维修资金管理办法，实行物业企业诚信等级评定和分级管理，多措并举解决好无物业管理小区问题。

　　居民住房保障力度进一步加大。投资35亿元，开工建设棚户区改造项目23个，建设房屋132万平方米，完成市政配套管线16.6公里。新建廉租房、公租房9513套、经济适用房2571套，4056户低收入家庭圆了“安居梦”。确定了全市首批公租房租金标准，扩大了申领保障性住房范围，廉租房、公租房、经济适用房实现并轨运行、互转保障。

　　就业和社会保障水平稳步提高。实现城镇新增就业9.8万人，创业带动就业1.56万人，应届高校毕业生就业率达88.9%，“零就业”家庭实现动态清零，城镇登记失业率为3.56%。继续实施重点紧缺工种、高校毕业生创业等免费专项培训，开展各类职业技能培训10.2万人次。转移农村富余劳动力就业6607人次。社会保险覆盖面进一步扩大，城镇各项社会保险参保人数净增8.6万人次，新型农村养老保险参保率97%，新型农村合作医疗参合率99.9%。社会保障水平进一步提高，连续10年调整企业退休人员基本养老金，月人均养老金达2376.6元。城镇职工基本医疗保险最高支付限额从17.4万元提高到30万元，城镇居民医疗保险大病费用报销实现上不封顶。

　　教育、卫生、体育等各项社会事业取得新进步。大力推进教育强区（县）工作，加快实施优质教育资源北扩工程，部分区（县）完成义务教育标准化建设和均衡发展任务。加大对普惠性民办幼儿园奖补力度，完成15所公办幼儿园建设任务。统筹以乌鲁木齐职业大学为骨干的教育资源，组建职业教育集团。落实“两免一补”等各类资助资金3.63亿元，惠及85.7万人次。全面落实各项医改任务，人均基本公共卫生服务经费补助标准达到70元。公办社区卫生服务中心（站）实现了基本药物制度全覆盖和基本药物“零差率”销售，惠及群众133.3万人次。疾病预防控制体系进一步完善，重点传染病同比下降5.59%。人口和计划生育政策全面落实，人口自然增长率为6.42‰。食品药品监管体制进一步完善，群众饮食用药安全不断强化。第十三届全国冬运会冰上运动中心场馆项目全面建成。建成乌鲁木齐县全民健身活动中心和米东区体育公园。深入开展双拥共建活动，军政军民团结进一步巩固，国防和军队建设不断加强。新闻出版、防灾减灾、外事、工商、质监、气象、地震、人防、地方志、档案工作取得新进展。老龄、妇女儿童、残疾人和红十字事业取得新成绩。

　　（四）现代化国际城市建设迈上新台阶

　　城市规划建设管理水平不断提高。乌鲁木齐城市总体规划获国务院批准实施。按照老城区改造提升规划要求，从严控制人口和建筑密度，增加公共活动空间和服务设施，持续改善老城区人居环境。加快高铁、会展、白鸟湖、城北新区、古牧地等新区建设。城市供水瓶颈改造、排水管网三期加快推进，新增供排水管网100公里。4个110千伏输变电项目建成送电，19个输变电项目开工建设。完成火车南站站前广场改造等工程，建成BRT虹桥停保场。深入开展环境综合整治，打造“田”字路沿线景观。大浦沟固体废弃物综合处理项目建成并投入使用，循环经济园区加快建设。建成各类垃圾房（站）530座，免费开放公厕777座，生活垃圾无害化处理率达92.5%。城区道路清扫保洁率达100%，冬季清雪做到“即下即清、雪停路净”，达到国内领先水平。

　　现代综合交通枢纽作用逐步强化。兰新高铁全线开通运营，高铁新客站站房主体工程完工。开通乌鲁木齐至石河子、奎屯、克拉玛依城际列车，开行中欧新疆西行货运班列52列。乌鲁木齐航空公司正式组建运营，开通疆内外航线6条。地铁1号线开工建设，公铁联运客运站项目稳步推进，三屯碑快速公交枢纽基本完工，绕城高速公路东线全面开工建设。实施了22个重点道路交通项目，西虹路—西山高架二期、克南高架东延、仓房沟路等道路建成通车，城市快速骨干路网更加完善。

　　大气污染治理成效显著。继续巩固“煤改气”成果，实施16个大气污染治理项目，完成4家公用电厂、5家自备电厂脱硝和脱硫除尘设施改造升级，完成5家重点企业污染治理项目。继续推进供热系统节能改造，完成燃气锅炉烟气余热冷凝改造1600蒸吨，改造老旧供热管网337公里。实现既有建筑节能改造360万平方米、供热计量改造1058万平方米。成为全国首批中德低碳生态试点示范城市。“煤改气”工程获2014年中国人居环境范例奖。米东区大气污染治理取得阶段性成果，关停污染企业300家，实施清洁能源改造企业119家。首府环境空气质量持续改善，主要污染物浓度持续下降，全年空气优良天数达310天，优良率达85%。

　　生态建设取得新突破。新增绿化面积3.5万亩，新建小游园及街旁绿地100个，小水面16处，绿道8条。实施森林抚育1.5万亩、封育2万亩、大苗基地2529亩。启动环城生态圈规划建设，开工建设天山区东部山丘生态休闲公园、赛马场十七户湿地公园。创建“花园式”单位20个、花园社区5个，新增绿化合格单位25个。荣获全国首批创建生态文明典范城市称号。推进城市饮用水源地环境保护，完成乌拉泊水源地流动源治理，红雁池水库调整为饮用水水源保护区。主城区再生水回用率达40%，成为“全国节水型社会建设示范区”。

　　（五）改革开放实现新突破

　　重点领域改革取得新进展。在全疆率先公布市本级部门行政审批“权力清单”，取消、下放和调整行政审批事项45项，占全部行政审批事项的30%；下放市级管理权限90项，占现有市级管理权限的32.26%。出台激发民间投资活力的政策措施，推出放宽准入等“12条新政”。深化“营改增”试点改革，为企业减负10.2亿元。混合所有制经济实现新发展。稳步推进市级国有经营性资产统一监管。不断创新融资方式，企业债券、私募债券、保险债券等非银行贷款方式融资到位资金创历史新高。就业、教育、医药卫生等社会事业领域改革稳步推进，全面深化改革实现良好开局。

　　对外开放进一步扩大。第九届丝绸之路市长论坛暨2014年丝绸之路经济带城市合作发展论坛取得丰硕成果，来自25个国家48个中外城市代表共同发表了乌鲁木齐宣言，共同发起了建立丝绸之路经济带城市联盟的倡议。制定丝绸之路经济带核心区“五大中心”建设实施方案，启动医疗康复服务和文化科教中心项目，“五大中心”建设实现良好开局。对中亚主要贸易伙伴出口大幅增长，服务贸易进出口增长2.1倍，对外直接投资总额16.9亿美元，增长31.8倍。哈萨克斯坦、格鲁吉亚等境外产业园区建设步伐加快。完成招商引资项目305个，引进区外到位资金626亿元，增长21.2%；实际利用外资2.6亿美元，增长15.7%。

　　（六）现代文化建设步伐不断加快

　　围绕构建社会主义核心价值体系，广泛开展主题宣传教育，扎实推进公民道德建设，开展“道德模范”、“美德少年”、“最美乌鲁木齐人”评选活动。深化服务行业、窗口单位和旅游景区文明服务达标活动，推进文明城区、文明单位、文明社区（村）和文明户创建工作，成功创建为全国文明城市提名资格城市。公共文化服务体系不断完善，市文化中心“六馆”建设稳步推进，一批区（县）文化场馆和社区（村）综合文化服务中心相继建成。大力实施文化惠民工程，新建公益电影固定放映场所50个，放映公益电影9216场次，举办各类群众性文化活动3700余场次，惠及各族群众482万人次。推动广播电视进社区工程，为4.72万户自建房群众安装有线电视。向近8万户少数民族流动人口家庭赠送《乌鲁木齐晚报》。鼓励文艺精品创作，推出大型现代秦剧《军医庄仕华》、大型双语报告文学集《首府的阳光》。设立文化产业发展专项资金，加快文化创意产业园建设，新增自治区级、市级文化产业示范基地26家。成功举办首届文化产业创意大赛。

　　（七）政府自身建设全面加强

　　扎实开展以“为民务实清廉”为主题的党的群众路线教育实践活动，聚焦政治上不够坚强和“四风”突出问题，狠抓整改落实和建章立制，开展24个方面36项专项整治，集中解决了一批作风上的突出问题，“四风”积弊得到有效整治，大型会议活动、文件简报数量进一步压缩，各类评比达标表彰活动大幅减少，“三公”经费支出明显下降，干部精神面貌焕然一新，让各族群众看到了新变化新气象。自觉接受人大法律监督和政协民主监督，办理自治区、市人大代表议案、建议和政协提案605件，办复率100%。提请市人大常委会审议地方性法规草案2件，制定政府规章4件。加强政务公开和网络问政，受理市长专线1.83万件，办结率98.89%；市长信箱1628件，办结率100%；网络问政13310项，回复率99.1%。不断深化依法行政，深入推进“六五”普法工作。行政监察、审计监督工作扎实推进，严肃查处了一批违法违纪案件，预防和惩治腐败成效明显。

　　各位代表，回顾2014年的工作，我们深深感到：所有这一切，是自治区党委、自治区人民政府和市委坚强正确领导的结果，是全市各族人民共同努力和各方面大力帮助支持的结果。在此，我代表市人民政府，向全市各族干部群众，向驻乌部队、武警官兵、公安民警、兵团职工、援乌干部，向人大代表、政协委员、各民主党派人士，向所有关心支持首府改革、发展与稳定的各界朋友，表示衷心的感谢并致以崇高的敬意！

　　在总结成绩的同时，我们也清醒地看到前进道路上面临的困难和问题：一是反恐维稳形势极其严峻，针对“三股势力”破坏的新动向新特点，严打严防措施还需进一步强化。二是民族团结教育不够深入，“去极端化”任务依然艰巨。三是宏观经济环境复杂多变，经济持续快速发展的压力较大，现代化国际城市建设力度需要进一步加大。四是改善民生离群众的期望还有差距，基本公共服务能力有待进一步提升。五是全面深化改革和推进依法治市任务紧迫，对各级领导干部改革创新能力提出了新考验。对此，我们将在今后的工作中采取措施切实加以解决。

　　2015年主要工作

　　2015年是“十二五”规划的收官之年，也是首府率先实现全面建成小康社会的决胜之年；是全面推进依法治市的开局之年，也是全面深化改革的关键之年，我们还将迎来自治区成立60周年。做好今年各项工作，对加快推进首府社会稳定和长治久安具有十分重要的意义。

　　2015年全市工作的总体要求是：高举中国特色社会主义伟大旗帜，认真贯彻落实党的十八大、十八届三中、四中全会、中央经济工作会议、第二次中央新疆工作座谈会和自治区党委八届六次、七次、八次全委（扩大）会议、经济工作会议、稳定工作会议，以及市委十届十一次全委（扩大）会议精神，坚持围绕社会稳定和长治久安这个总目标，以打击防范“三股势力”、加强民族团结、促进宗教和谐、遏制宗教极端思想渗透蔓延、“去极端化”为重点，全力维护社会大局稳定；坚持稳中求进、改革创新总基调，以提高经济发展质量和效益为中心，把转方式调结构放到更加重要位置，主动适应经济发展新常态；坚持营造“四大环境”、建设“五大中心”、打造“六大产业基地”、构建“七大组团”的基本思路，全面深化改革，全面推进依法治市，全面建成小康社会，努力建设全疆团结和谐、繁荣富裕、文明进步、安居乐业的首善之城。

　　2015年经济社会发展的主要预期目标是：实现地区生产总值增长10%；全社会固定资产投资增长18%；社会消费品零售总额增长11%；全口径进出口贸易总额增长10%；地方财政收入增长10%，其中公共财政预算收入增长10%；城镇居民人均可支配收入增长13%；农牧民人均纯收入增长15%；居民消费价格总水平涨幅控制在4%以内；新增城镇就业7.5万人，城镇登记失业率控制在3.7%以内，全面完成“十二五”节能减排目标。实现上述目标任务，我们要重点做好以下工作：

　　（一）紧紧围绕总目标和着眼点着力点，坚定不移维护社会大局持续稳定

　　当前，打击“三股势力”、严防暴恐活动，是维护社会大局稳定的首要任务。我们要坚决贯彻中央、自治区党委和市委一系列重大决策部署，坚持“打击的一手要硬，教育疏导的一手也要硬”，保持主动进攻态势，围绕总目标、案件汲教训、查找薄弱点、工作抓落实，确保实现“三个坚决防止”目标，着力营造和谐稳定的社会环境。

　　一是狠抓严打严防各项措施落实。坚持主动进攻，打准打狠打出声威，打早打小打苗头，始终保持严打高压态势，不断深化严打暴恐专项斗争。坚持以防为先、立足于防，进一步加强重点区域、要害部位、重点目标安全防范，强化点、线、面相结合的巡逻防控网络，构建严密的反恐防范体系。坚持问题导向，继续深入查找和堵塞防范漏洞，坚持不懈防范风险点、加强薄弱点，及时解决各类隐患问题。进一步健全群防群治工作机制，确保所有安全防范措施一一落实到位，形成全社会人人有责、积极参与、齐抓共管的局面。

　　二是深入推进“去极端化”工作。认真落实自治区党委11号和28号文件精神，学会用好“五把钥匙”，坚持疏堵结合、多措并举，认真落实《乌鲁木齐市公共场所禁止穿戴蒙面罩袍的规定》，提倡世俗化生活方式，坚决遏制宗教极端，淡化宗教氛围。进一步加大“三非”治理力度，坚决取缔地下讲经、学经活动，全力查缴非法出版物和宣传品，坚决切断宗教极端思想的传播渠道。按照“到人、管用、有效”的要求，切实强化正面宣传引导，综合运用电视电影、报纸网络、广播喇叭、手机信息等宣传手段，深入推进“去极端化”和“一反两讲”宣讲活动进机关、进学校、进企业、进农村、进社区、进家庭，实现教育宣讲全覆盖。继续向少数民族流动人口家庭赠送《乌鲁木齐晚报》。加强对流动人口的服务管理，在全市推广沙区平顶山社区在服务流动人口方面的典型经验，促进流动人口尽快融入现代城市生活，成为维护首府稳定的支持者和实践者。切实加强意识形态领域阵地建设，增强对意识形态领域的控制力、引导力。

　　三是切实加强民族团结促进宗教和谐。坚定不移地贯彻落实好党的民族政策，大力开展民族团结宣传教育活动、民族团结进步创建活动和多层次、多渠道、多形式的民族团结联谊交流活动，进一步密切关系、融洽感情，不断巩固全市各族人民大团结的良好局面。推动各民族嵌入式小区示范工程建设，促进各族群众形成相互往来、邻里守望的友好关系。加强青少年民族团结教育引导工作，把民族团结教育贯穿到学校、家庭、社会等各个方面，让各族青少年从小玩在一起、学在一起、成长在一起，使民族团结蔚然成风。全面贯彻落实党的宗教政策，积极引导宗教与社会主义社会相适应。依法加强宗教事务管理。继续扎实开展“双五好”创建活动，影响和带动广大信教群众确立正信、抵制极端，营造宗教界爱国感恩、崇尚和谐的良好氛围。

　　四是进一步加强基层基础。围绕打牢“三个基础”，加强以街道（乡镇）、社区（村）为重点的基层组织建设，巩固和提升社区“十有”条件，赋予社区更多的资源和手段，让社区组织在维护稳定和服务群众中发挥更大作用。继续实行优秀公岗人员纳编、逐年提高收入水平等政策，不断加强基层干部队伍建设。深入推进社区“四化”管理，提升基层维稳防控管理能力。继续深入开展“访惠聚”活动，全力做好面对面的群众宣传教育工作，为群众办实事，帮群众解难题，与群众交朋友，最大限度地争取和凝聚民心。

　　（二）加快“六大产业基地”发展，积极构建现代产业体系

　　主动适应经济发展新常态，加快转方式、调结构，围绕打造“六大产业基地”，坚持发展高端产业，突出重大项目，壮大实体经济，着力构建具有市场竞争力的现代产业体系，增强经济发展动力和活力。

　　一是大力发展先进制造业。以产业集群规划布局为引导，加快园区转型升级和企业聚集、产业集群，全面推动产业基地向差异化、特色化方向发展。先进装备制造业基地启动汽车零部件专业化生产企业引进计划；高新技术产业基地加快促进光伏产业、电子新材料产业壮大；战略性新兴产业基地重点发展现代煤化工、石油石化精细化工等产业集群，确保高端产业总量和实力快速攀升。积极引进和建设石油化工产业链延伸项目，确保“十二五”末形成千万吨炼油、百万吨化肥、百万条子午线轮胎生产能力。促进钢铁产业向特种钢、优质钢及板材钢等产品转型。加快推进乌鲁木齐国际纺织品服装商贸中心建设，促进纺织服装产业园区发展，提升纺织产业竞争力。加快推进伊泰540万吨煤基多联产、金风200兆瓦风电场等18个重点工业项目建设。促进工业化与信息化深度融合，协调推进智慧城市、云计算产业基地、新疆软件园及三大数据库等信息产业重点项目。加快产业创新体系建设，扶持和培育市级工程技术研究中心6家，新增产学研联合开发示范基地2个、高新技术企业、创新型企业30家。全力推进中小企业公共服务、信用服务平台和科技孵化器建设，促进中小微企业开拓市场、创建品牌、扩大就业。

　　二是加快推进服务业优化升级。深化现代服务业综合改革试点，加快会展片区现代服务业基地建设。大力发展信息服务、电子商务、文化创意等新兴服务业，优先发展现代金融、商贸物流、会展商务等生产性服务业，促进旅游、餐饮、健康服务、养老服务等生活性服务业转型升级。推进金融城、新疆国际会展中心二期建设。促进重点领域消费，打造多点支撑的消费增长格局。做好重大展会服务保障工作，全年举办展会100个以上。加快城南经贸合作区规划建设，2015年启动起步区道路等基础设施建设，积极开展合作区招商引资工作。加快推进南山旅游基地规划建设，深度整合乌鲁木齐县与达坂城区旅游资源，抓好特色旅游项目开发建设，加快向国内一流旅游景区目标迈进。

　　三是促进城乡一体化发展。以增加农牧民收入为核心，继续调整农业产业结构，大力发展设施农业、节水农业和休闲观光农业。新（改）建设施农业6000亩，新（改）建养殖小区50个，流转农村土地15万亩。推进国家农业科技园区建设，加快建立完善园区管理体制与运行机制。完善农产品质量安全检测体系，新增农产品质量安全检（监）测点40个。大力扶持农民合作社发展，培育农产品知名品牌，做精“农家乐”、“牧家乐”，千方百计促进农牧民增收。继续落实好支持农业农村工作实施方案，全面完成“两居”工程建设任务，进一步完善水利、道路等基础设施，加快新型城镇化建设步伐。加强农村生态建设，全面实施农村环境综合整治，完成基本草原划定和新一轮退耕还林还草任务。

　　（三）全面深化改革，进一步增强发展内生动力

　　深入推进重点领域改革。建立健全三级国有资产监管运营模式，逐步实现由管资产向管资本转变，由审批制向报备制转变。大力发展混合所有制经济，推动市属国有企业参股央企、自治区企业和具有发展潜力的其他所有制企业。加快完善企业监管信息公示平台，推进企业诚信建设。深化投融资体制改革，拓宽投融资渠道，设立产业投资基金或投资引导基金，鼓励民间资本发起设立产业投资基金和股权投资基金，积极探索推行政府和社会资本合作（PPP）等多种投融资模式。推动非公有制企业和社会资本广泛参与基础设施、市政环卫、公共服务等领域投资。深化预算管理制度改革，推行政府购买服务。以公立医院改革为重点，深化医药卫生体制改革，不断提高医疗服务质量。继续推进城市管理权限下放，做实区（县）完全责任型政府。加快区、市两级行政服务中心（公共资源交易中心）建设，创新行政审批服务方式，推行“一个窗口对外、一条龙服务”，努力把我市建成西部“审批事项最少、效率最高、服务最优”的城市之一。

　　加快社会治理创新。以建设和谐社区为重点，进一步完善社区网格化管理和社会化服务功能。加大信访工作力度，不断健全社会矛盾纠纷预防化解机制，把矛盾纠纷化解在基层和源头。全面推进安全生产标准化、隐患排查治理体系建设，持续深入开展“打非治违”专项行动和重点行业领域专项整治行动，坚决防止重大安全生产事故发生。积极支持国防和军队革命化现代化正规化建设，不断完善国防动员体系，提高后备力量建设质量。加强军政军民团结，争创全国双拥模范城“八连冠”。

　　（四）进一步扩大开放，加快建设丝绸之路经济带核心区“五大中心”

　　抓住国家推进丝绸之路经济带建设的战略机遇，加快对外开放环境建设，积极承接产业转移，不断增强区域中心城市的开放功能，全面提升我市在丝绸之路经济带的地位和影响力。

　　全力打造丝绸之路经济带核心区“五大中心”。编制完成国民经济和社会发展第十三个五年规划，加快推进“五大中心”专项规划编制。围绕打造交通枢纽中心，加快构建“三环十五射”的路网骨架，完善乌昌石交通网络体系；积极推进地铁1号线项目，完成2号线前期工作；全面建成高铁新客站并投入使用，开通至吐鲁番、库尔勒、准东的城际铁路；推动乌鲁木齐国际机场改扩建工程，积极支持机场申请72小时过境免签政策，争取开通至中亚、西亚、南亚、欧洲等地区航线。围绕打造商贸物流中心，继续推进“五大物流园”建设。重点支持乌鲁木齐铁路货运站和集装箱中心站建设，积极推进建设中欧班列集结编组中心和进出口货物分拨中心，切实提升乌鲁木齐在国际铁路货运物流中的始发、集结、编组、分拨能力，带动首府物流业快速发展。围绕打造金融服务中心，积极争取国内外金融、证券和中介服务机构在乌设立经营机构、业务代理机构，开展跨境金融业务。围绕打造科教文化中心，鼓励和支持丝绸之路沿线高校、科研院所、企业联办研发机构联合承担国家重大科技项目，发展多种形式的科技合作与交流；加快推进市文化中心“六馆”建设，办好丝绸之路国际文化产业博览交易会。围绕打造“医疗服务中心”，进一步完善医疗设施，加快建立医疗服务合作联合体，建设一批具有国际服务水平的重点医疗机构。继续利用好中国—亚欧博览会、丝绸之路经济带城市合作发展论坛等平台，不断提升我市在丝绸之路经济带中的地位和影响力。

　　全方位扩大对外开放。积极申报亚欧经贸合作试验区，加快综合保税区和乌鲁木齐陆路港建设。大力吸引国内外具有先进技术和管理水平的企业，实际利用外资和引进区外到位资金增长10%以上。做大一般贸易、壮大加工贸易、优化边境贸易，进一步扩大高新技术产品、地产品出口规模。积极发展服务贸易和服务外包产业。支持企业开拓国际市场，鼓励企业开展跨国经营，建立营销服务网络。支持兵团十二师充分发挥“四个作用”，探索兵地融合发展新模式。

　　（五）着力保障和改善民生，让发展成果惠及各族群众

　　坚持民生优先，群众第一，继续实施“民生改善年”活动，办好今年改善民生100件实事，不断改善各族群众生产生活条件。

　　一是切实做好就业再就业工作。鼓励创业带动就业，实现创业就业1.6万人。加大高校毕业生和城乡困难人员就业工作力度，支持各类企业吸纳本地劳动力就业。加强就业和创业培训，实施重点紧缺工种、高校毕业生创业等免费培训计划，开展各类职业技能培训10.5万人次。健全公共就业服务体系，加快推进市级中心人力资源市场和50个基层公共就业“一站式”服务大厅建设。

　　二是加快完善统筹城乡的社会保障体系。扩大社会保险覆盖面，努力实现应保尽保。推动城乡养老保险制度衔接，实现城镇职工和城乡居民养老保险制度相互流转。启动城乡居民基本医疗保险统筹工作，提高筹资和保障水平。扩大生育保险覆盖范围，将灵活就业人员和城镇居民纳入生育保障。更加关注低收入群体、困难家庭生活，完善社会救助体系，提高城乡低保标准，构筑民生保障底线。加强粮、油、肉、菜等生活必需品市场监测和监管，健全应急物资储备体系，稳定居民生活必需品价格。

　　三是全面推进教育医疗卫生事业发展。全力实施教育强区（县）工程，推进义务教育学校捆绑式发展，提高义务教育均衡发展水平。加快完善学校基础设施，力争完成一中、十三中新校区建设。大力发展学前教育，加快公办幼儿园建设，扩大普惠性幼儿园覆盖面。推动职业教育集团化办学，培养更多技能型人才。提高“双语”教育水平，完善特殊教育体系。严格落实安全防范责任，打造平安校园。完善城乡医疗卫生服务体系，抓好疾病预防控制工作。加强流动人口计生服务，提高出生人口素质。提升食品药品监管整体水平，切实保障食品药品安全。落实全民健身计划，广泛开展群众性体育活动。高标准改造红山体育馆，启动新体育中心建设。全力备战全国第十三届冬季运动会。促进妇女儿童、残疾人和红十字事业，推动统计、外事、工商、质监、人防、地方志、档案等各项社会事业蓬勃发展。

　　四是加快棚户区改造和保障性住房建设。开工建设棚改房6000套、60万平方米，新建各类保障性住房1.15万套。坚持棚改房、保障性住房与市政配套设施、公共服务设施同步建设、同步交付使用，确保居民安置和居住条件改善。加快完善住房保障体系，完善保障房分配和使用监管机制，制定“三房合一”政策，探索吸纳社会房源用于公租房，解决好低收入群体住房问题。

　　（六）推进城市空间转型升级，加快建设现代化国际城市

　　认真落实城市总体规划，坚持“南控北扩、先西延后东进”呈扇面发展的战略方向，坚持多中心、组团式的城市空间布局，加快形成市域住房、生活、发展空间的合理结构，着力推进宜业宜居的现代化国际城市建设。

　　一是强化规划龙头作用。坚持以城市规划为引领，实现“三规合一”。进一步优化城市布局、增强城市功能、改善生态环境、提升城市品质，梳理各层次规划的衔接修订工作，深化完善老城区改造提升规划，优化调整乡镇（村）规划，实现中心城区控制性详细规划全覆盖。强化规划实施管理和执法监察工作，切实维护规划的严肃性。

　　二是加快推进新区建设和老旧城区改造。以“七大组团”建设为重点，大力推进高铁、会展、白鸟湖、城北新区、古牧地、城南经贸合作区等新区建设，推进学校、医院及各类城市综合体项目加快建设，打造产城融合、宜业宜居的现代化新城区。持续改善老城区人居环境，重点实施二道桥等片区改造提升工程。

　　三是强力推进重大基础设施建设。启动东二环快速路、新医路西延征收和阿勒泰路高架桥、珠江路立交节点改造、城北主干道机场高速互通立交等新建项目，争取绕城高速公路东线全线通车。推进750千伏西山、甘泉堡变电站等城乡供电体系建设。加快西山水厂改扩建、头屯河饮水管线项目，完成观园路片区供水、大西沟管线饮水工程，新增供排水管网120公里。加快“公交都市”建设，建成BRT6号线，开工建设头屯河综合客运枢纽、南山旅游基地综合客运枢纽。

　　四是提高城市精细化管理水平。围绕创建国家卫生城市和环保模范城市，以庆祝自治区成立60周年为契机，持续开展环境综合整治，营造干净整洁、清新靓丽的城市环境。完善城市管理标准综合评价体系，实施环境整治综合考核，推进网格化、数字化和分级分类管理，实现城市管理由粗放型向精细化转变。推进生活垃圾焚烧发电等废弃物处理项目，逐步扩大生活垃圾分类收运试点。推广使用小型设备开展精细化作业，提高机械化保洁和清雪覆盖率。完善城市公共安全应急保障体系，进一步提高城市安全运行保障能力。

　　（七）加快生态文明建设，不断改善人居环境

　　牢固树立生态文明理念，推进绿色发展，加大城乡环境保护力度，实行严格的生态保护措施，不断提升人居环境质量。

　　一是不断巩固大气污染治理成果。严格落实“八个一律”措施，强化联防联控机制，确保首府空气质量优良率达到85%以上。加快实施南部集中供热工程和配套管网工程，充分利用风电场弃电在达坂城区和乌鲁木齐县示范推广供热项目建设。完成既有建筑供热计量及节能改造150万平方米，老旧供热管网改造180公里。推广电动汽车、电采暖等清洁能源替代应用。加大扬尘污染治理力度，散装物料储运实现全封闭。推进米东区工业污染源治理，关停淘汰落后产能企业480家。强化机动车尾气治理，加快淘汰“黄标车”。

　　二是深入推进以园林绿化为主的生态建设。围绕创建国家生态园林城市目标，继续推进植树造林和园林绿化工作，新增绿化面积3.5万亩。启动东绕城生态圈项目建设，实施15个规模绿化公园项目，建成小游园小绿地100个、小水面8处。严守水资源“三条红线”，加强水源地保护，从严查处污染地下水行为，保障城市饮水安全。深入推进柴窝堡湖、盐湖生态治理，实施头屯河、水磨河流域专项整治，力争将大西沟、乌拉泊、红雁池水库生态环境保护纳入全国湖泊生态环境保护试点。加快推进虹桥、雅山污水处理厂提标改造，不断提高再生水利用率。继续关停一批破坏生态环境、存在安全隐患的小煤矿。

　　三是加大节能减排力度。以列入国家第二批低碳试点城市为契机，落实国家节能减排财政综合示范城市各项政策，严格污染物排放总量控制和能源消费总量控制。强化重点工业企业节能监察，完成52家工业企业能源体系建设工作。完善公共建筑节能监管体系，严格执行新建公共建筑节能50%、居住建筑节能75%的强制性标准，支持开展绿色建筑、低能耗建筑、住宅产业化示范项目。建成餐厨垃圾资源化利用和无害化处理二期工程，积极申报大浦沟固废处理示范园为自治区循环经济试点园区。全面贯彻实施最严格的耕地保护、水资源管理和环境保护制度，推进节约集约利用资源。

　　（八）以现代文化为引领，加快建设首善之城

　　坚持用社会主义核心价值观引领社会思潮，不断筑牢各民族共同的精神家园。大力传播现代文明理念和行为方式，引导各族群众牢固树立国家意识、公民意识、法治意识，形成知荣辱、讲正气、尽义务、懂感恩的文明风尚。深入推进全国文明城市创建工作，实施文明创建细胞工程，扎实推进公民道德建设，不断提高市民文明素质，建设以人为本、人心向上、有品位有特色、充满活力的“首善之城”。健全城乡四级公共文化服务体系，推进区（县）文化场馆建设，加强社区（村）文化室标准化建设和规范化管理。继续抓好社区公益电影放映和广播电视“村村通”、“户户通”工程，提高少数民族语言广播电视覆盖率。健全政府购买演出机制，深入开展文化惠民演出活动，创作一批文艺精品，丰富各族群众文化生活。推动特色文化街区建设，培育壮大骨干文化企业，促进创意设计、数字影音、网络传媒等文化产业发展。

　　（九）强化政府职能转变，着力建设法治政府

　　我们要按照党的十八届四中全会和自治区党委八届八次全委（扩大）会议提出的，深入推进依法行政、加快建设法治政府的决策部署，巩固深化党的群众路线教育实践活动成果，切实抓好各项工作的落实，不断提高政府公信力和执行力，全面建设法治政府。

　　一是坚持依法行政。依法全面履行政府职能，推进机构、职能、权限、程序、责任法定化，坚持法定职责必须为、法无授权不可为。继续深化行政审批制度改革，推行政府“权力清单”制度。健全规范性文件、重大决策合法性审查机制，建立法律顾问制度。进一步规范行政执法程序，加强执法监督检查，全面落实执法责任制和执法过错责任追究制。健全政府议事规则，完善重大事项公众参与、事前论证、风险评估、合法性审查和集体讨论相结合的决策制度。涉及重大公共利益、人民群众切身利益的决策事项，都要向社会公开征求意见或举行听证会。严格执行市人大及其常委会的决议决定，自觉接受人大工作监督、法律监督和人民政协民主监督，认真听取民主党派、工商联、无党派人士和人民团体的意见建议，高度重视社会监督和舆论监督。

　　二是坚持廉洁高效。按照“三严三实”和“四强”标准要求，狠抓政风行风建设，持续治理“庸懒散拖贪”，不断提高行政效能和服务水平。强化对政府职能部门履行职责情况的监督，加强行政监察和审计监督，加大行政问责力度。严格执行中央八项规定和自治区党委、市委十条规定，落实党风廉政建设责任制，健全廉政风险防控体系。加大违法违纪案件查办力度，旗帜鲜明地反对腐败，确保廉洁从政。深入推进政务公开，坚持以公开为常态、不公开为例外的原则，推进决策公开、执行公开、管理公开、服务公开、结果公开，方便群众了解政府工作，监督政府工作。

　　三是坚持真抓实干。一分部署，九分落实。紧紧围绕首府发展重大问题和市委各项决策部署，加强调查研究，察实情、出实招、办实事，不断增强理解力、落实力、执行力，推动工作落实。坚持“两个务必”，坚决整治“四风”，以实际行动保持同人民群众的血肉联系。坚持“只有努力才能改变，只要努力就能改变”，牢记使命，恪尽职守，夙夜在公，始终保持干事创业的热情、动真碰硬的豪情、奋发有为的激情，不断创造经得起实践、群众和历史检验的业绩，取信于民、造福于民。

　　各位代表，梦想承载希望，奋斗开创未来。让我们紧密团结在以习近平同志为总书记的党中央周围，在自治区党委、自治区人民政府和市委的坚强正确领导下，全力确保社会大局稳定，紧贴民生推进跨越发展，干在实处、走在前列、当好表率，为把首府建成全疆团结和谐、繁荣富裕、文明进步、安居乐业的首善之城而努力奋斗！
